# Supplementary material for: Mobile health supported real-time guidance and debriefing for newborn resuscitation: A pilot study of LIVEBORN feedback
Source: PLoS One. 2026 Jan 8;21(1):e0310606. doi: 10.1371/journal.pone.0310606 (PMC12782419; doi:10.1371/journal.pone.0310606)
Supplement: S1 File — (PDF) [file pone.0310606.s001.pdf]

The LIVEBORN Study:
An Integrated mHealth Strategy to Improve Newborn Resuscitation in Low- and Lower Middle-Income Countries

Protocol Version 3.0

September 28 2021

Funded by:
Eunice Kennedy Shriver National Institute of Child Health and Human Development
1R21HD103058-01

Principal Investigator: Jacquelyn K Patterson, MD, MPH
Assistant Professor of Pediatrics
Division of Neonatal-Perinatal Medicine
University of North Carolina at Chapel Hill

Co-Investigators:
Carl Bose, MD
Ben Chi, MD, MSc
Joar Eilevstjønn, PhD
Patricia Gomez, MPH
Ingunn Haug, MSc
Daniel Ishoso, MD, MPH
Eric Mafuta, MD, MPH, PhD
Helge Myklebust, BS
Antoinette Tshefu, MD, MPH, PhD

Protocol Signature Page

**Protocol Title:**	The LIVEBORN Study: An Integrated mHealth Strategy to Improve Newborn Resuscitation in Low- and Lower Middle-Income Countries
**Short Title:**	The LIVEBORN Study
**Protocol Version:**	3.0
**Version Date:**	September 28 2021

I, the Investigator of Record, agree to conduct this study in full accordance with the provisions of this protocol. I will comply with the provisions of this protocol, all requirements regarding the obligations of clinical investigators as fully outlined in the International Conference on Harmonization (Section E6(R2) Good Clinical Practice), local regulatory requirements, and the Investigator's Agreement, which I have also signed.

I have read and understand the information in the protocol and will ensure that all staff assisting in the conduct of the study are informed about the obligations incurred by their contribution to the study.

Investigator of Record Name: ______________________________

Investigator of Record Signature: ______________________________

Date: ______________________________

Version Tracking

Version	Date	Comments
1.0	January 12 2021	
2.0	February 2 2021	Details regarding usability evaluation
3.0	September 28 2021	Details regarding integrated strategy design with feasibility testing

Table of Contents

Abbreviations and Acronyms 6

Study Summary. 7

1 Introduction 9
1.1 Statement of the Problem 9
1.2 Background and Rationale 9

2 Study Objectives and Outcomes 10
2.1 Study Objective 10
2.2 Primary Outcomes 10
2.3 Secondary Outcomes 10

3 Methodology 11
3.1 Study Design 11
3.2 Formative Research 11
3.2.1 Study Sites 11
3.2.2 Study Population 11
3.3 Usability Evaluation 11
3.4 Integrated Strategy Design with Feasibility Testing 11

4 Study Procedures 11
4.1 Formative Research 12
4.1.1 Participant Recruitment 12
4.1.2 Participant Screening and Enrollment 12
4.1.3 Focus Group Discussions 12
4.2 Usability Evaluation 13
4.3 Integrated Strategy Design and Feasibility Testing 14

5 Data Management and Analysis 17
5.1 Formative Research 17
5.1.1 Data Security and Management 17
5.1.2 Sample Size 17
5.1.3 Analysis 17
5.2 Usability Evaluation 17
5.3 Integrated Strategy Design and Feasibility Testing 17
5.4 Dissemination of Findings 18

6 Ethical Considerations 18
6.1 Institutional Review Board Approval 18
6.2 Formative Research 19
6.2.1 Informed Consent 19
6.2.2 Potential Risks to Participants 19
6.2.3 Potential Benefits to Participants 19

6.3 Usability Evaluation 19
6.4 Integrated Strategy Design and Feasibility Testing 20

7 Study Organization, Coordination and Administration 21
7.1 Division of Responsibilities 21
7.1.1 UNC 21
7.1.2 KSPH 21
7.1.3 LGH 21
7.1.4 Jhpiego 21
7.2 Communication 22
7.3 Study Timeline 22

8 References 22

Abbreviations and Acronyms

**BMV**	bag mask ventilation
**DRC**	Democratic Republic of the Congo
**FIM**	Feasibility of Intervention Measure
**HBB**	Helping Babies Breathe
**KSPH**	Kinshasa School of Public Health
**LGH**	Laerdal Global Health
**LMICs**	low- and lower middle-income countries
**mHealth**	mobile health
**SUS**	System Usability Scale
**UNC**	University of North Carolina

STUDY SUMMARY

**Title**	The LIVEBORN Study: An Integrated mHealth Strategy to Improve Newborn Resuscitation in Low- and Lower Middle-Income Countries
**Short Title**	The LIVEBORN Study
**Methodology**	Usability study to design and evaluate LIVEBORN
**Purpose**	To develop a useable and feasible mobile health application to improve newborn resuscitation through feedback
**Study Participants**	Midwives and newborns in health facilities in the Democratic Republic of the Congo
**Study Activities**	<u>Formative research:</u> Focus group discussions with midwives in the Democratic Republic of the Congo<br><u>Usability evaluation:</u> Testing of LIVEBORN in simulated neonatal resuscitations with midwives in two rounds, with iterative refinement of LIVEBORN between rounds<br><u>Integrated strategy design with feasibility testing:</u> In collaboration with midwives, design of a strategy for incorporating LIVEBORN into clinical care for real-time guidance and one for debriefing; pilot feasibility testing of LIVEBORN during bedside resuscitations
**Study Sites**	Health facilities in the Democratic Republic of the Congo
**Coordinating Center**	UNC-Chapel Hill School of Medicine

Eligibility Criteria

Formative research

Inclusion criteria: Midwives with prior experience with NeoBeat and/or the beta version of the LIVEBORN app.
Exclusion criteria: Any condition which, in the opinion of LIVEBORN Study staff, would make participation unsafe.

Usability evaluation

Midwives employed at Bondo health center who meet the following criteria—

Inclusion criteria: Provision of newborn care at the time of birth during the course of their regular employment.
Exclusion criteria: Any work-place concerns regarding participation.

Integrated strategy design with feasibility testing

Midwives employed at Binza-Delvaux or Kingasani health facilities who meet the following criteria—

Inclusion criteria: Provision of newborn care at the time of birth during the course of their regular employment.
Exclusion criteria: Any work-place concerns regarding participation.

Outcomes

Primary Outcome

LIVEBORN usability

Secondary Outcomes

Feasibility of LIVEBORN observations
Feasibility of real-time guidance
Feasibility of debriefing

1 INTRODUCTION

This document is a protocol for a human research study. The LIVEBORN Study is to be conducted according to International Standards of Good Clinical Practice (International Conference on Harmonization guidelines www.ich.org), applicable government regulations, and institutional research policies and procedures. The *Eunice Kennedy Shriver* National Institute for Child Health and Human Development is funding this research.

1.1 STATEMENT OF THE PROBLEM

Almost one million newborns die each year from failure to breathe at birth. Nearly all of these deaths occur in low and lower-middle income countries (LMICs). These deaths result when life-saving bag mask ventilation (BMV) is delayed or interrupted. Simulation-based training alone is insufficient to ensure effective BMV. Scalable, complementary strategies are required to ensure timely and continuous BMV. There is strong scientific premise for improving BMV at the bedside using feedback strategies. Mobile health (mHealth) technology could enable implementation of feedback strategies in LMICs.

1.2 BACKGROUND AND RATIONALE

Respiratory depression causes most newborn deaths in the first 24 hours after birth. One million newborns die on their day of birth each year, accounting for one third of all newborn deaths.[1] Ninety percent of these deaths are from failure to breathe at birth (i.e., respiratory depression).[2] Nearly all of these deaths occur in LMICs.

Effective BMV at birth reduces newborn mortality. Basic resuscitation practices reduce death from respiratory depression. Among these practices, BMV has the greatest impact on mortality. To be effective, BMV must be timely. Resuscitation algorithms recommend initiating BMV of non-breathing newborns within 60 seconds after birth.[3,4] Delayed BMV increases the risk of death: for every 30-second delay in BMV, the risk of death increases by 16% in LMICs.[5,6] Effective BMV is also continuous. Resuscitation algorithms recommend continuous ventilation until spontaneous breathing begins. Interrupted BMV increases the risk of death by 75% in LMICs.[7]

Simulation-based training strategies are insufficient to ensure mastery of BMV. A common strategy to improve basic resuscitation practices, including BMV, is simulation training using a resuscitation algorithm such as Helping Babies Breathe (HBB).[8] HBB training reduces perinatal mortality. However, these reductions are infrequently sustained due to a decline in knowledge and skill over time.[8,9] A variety of complementary strategies have been investigated to mitigate this decline. These strategies include refresher training, frequent simulation practice, simulation with feedback, high-fidelity simulation, and teamwork training.[10-17] mHealth strategies have also targeted knowledge and skill decline with automated methods for testing knowledge, logging simulation practice and rehearsing skills through virtual reality.[18-20] Despite improved retention of knowledge and skill with several of these strategies, performance of BMV in LMICs remains ineffective. BMV is delayed in up to 78% of resuscitations in LMICs, with a mean initiation time of as long as 220 seconds after birth.[21,22] BMV is intermittent rather than continuous in 50% of resuscitations in LMICs.[23]

Improving BMV is critical to reduce newborn death in LMICs such as the Democratic Republic of the Congo (DRC). In 2018, the newborn mortality rate in the DRC was 28.3 per 1,000 live births.[24] Since 2005, the newborn mortality rate in the DRC has declined by an average of only 0.6 deaths per 1,000 live births per year. At this pace of mortality reduction, the DRC will not meet the 2030 Sustainable

Development Goal of 12 per 1,000 live births until after 2050.[25,26] In response, the DRC Ministry of Health and USAID are implementing HBB training with frequent simulation practice in 178 health zones over the next six years.[27] This training is likely to reduce mortality. However, reductions will be limited unless complementary, scalable strategies are developed to ensure effective BMV.

There is strong scientific premise for improving BMV with feedback strategies. Feedback on cardiopulmonary resuscitation for health professionals improves their performance.[14,15,28-35] Two feedback strategies for improving performance of BMV during simulation have demonstrated effectiveness: *real-time guidance* (feedback during practice) and *debriefing* (feedback after practice).[10-13,36-39] In high-income countries, these strategies are typically implemented at the bedside using expert clinicians and detailed data on resuscitation events. Feedback in LMICs is likely to have similar and significant impact. However, lack of both expert clinicians and detailed data are significant barriers to bedside feedback in LMICs. mHealth technology could enable the implementation and evaluation of these strategies in LMICs.

We will augment simulation-based training with bedside feedback using an mHealth app called LIVEBORN. We will develop LIVEBORN, an mHealth app that delivers automated feedback during newborn resuscitations. Our new heart rate monitor, NeoBeat, will be the centerpiece technology for the creation of this mHealth app. LIVEBORN is technology-appropriate for low-resource settings, relying on offline in-app delivery with Bluetooth streaming from battery-operated NeoBeat. If feedback with LIVEBORN is effective, it will improve BMV and reduce newborn mortality in LMICs.

2 STUDY OBJECTIVES AND OUTCOMES

2.1 STUDY OBJECTIVE

Our objective is to reduce newborn mortality by improving BMV in LMICs through bedside feedback using an innovative mHealth app called LIVEBORN.

2.2 PRIMARY OUTCOMES

Our primary outcome is LIVEBORN usability, defined using a previously validated metric for evaluation of technology called the System Usability Scale (SUS).[40] A score ≥68 will indicate sufficient usability.

2.3 SECONDARY OUTCOMES

Our secondary outcomes are related to the feasibility of using LIVEBORN during bedside resuscitations:

- Feasibility of LIVEBORN observations, defined as ≥50% of resuscitations observed
- Feasibility of real-time guidance, defined as a median Feasibility of Intervention Measure (FIM)[41] score >12
- Feasibility of debriefing, defined as ≥50% of observed BMV events having debriefing completed

3 METHODOLOGY

3.1 STUDY DESIGN

This is a usability study to design and evaluate LIVEBORN. We will develop LIVEBORN through a scientifically rigorous process involving formative research and technical development. We will evaluate usability of LIVEBORN during two rounds of simulated resuscitations with midwives, with iterative refinement of LIVEBORN between rounds. In collaboration with Congolese midwives, we will design integrated mHealth strategies for both real-time guidance and debriefing. Finally, we will evaluate feasibility of LIVEBORN in a pilot feasibility test in health facilities with midwives.

3.2 FORMATIVE RESEARCH

3.2.1 Study Sites

Formative research will be conducted in three health facilities in Kinshasa, DRC.

3.2.2 Study Population

Midwives who provide newborn care and meet the following criteria will be eligible for participation:

Inclusion criteria:

Midwives who have previously used NeoBeat and/or the beta version of the LIVEBORN app through prior participation in the NeoBeat Study or the Liveborn Usability Study.

Exclusion criteria:

Any work-place concerns regarding participation.

3.3 USABILITY EVALUATION

3.3.1 Study Sites

The usability evaluation will be conducted at Bondo health facility in Kinshasa, DRC.

3.3.2 Study Population

Midwives employed at Bondo who meet the following criteria will be eligible for participation:

Inclusion criteria:

Provision of newborn care at the time of birth during the course of their regular employment.

Exclusion criteria:

Any work-place concerns regarding participation.

3.4 INTEGRATED STRATEGY DESIGN WITH FEASIBILITY TESTING

3.4.1 Study Sites

Integrated strategy design with feasibility testing will be conducted at Binza-Delvaux and Kingasani health facilities in Kinshasa, DRC.

3.4.2 Study Population

Midwives employed at Binza-Delvaux or Kingasani who meet the following criteria will be eligible for participation:

Inclusion criteria:

Midwives who provide newborn care at the time of birth during the course of their regular employment.

Exclusion criteria:

Any work-place concerns regarding participation.

4 STUDY PROCEDURES

We will design LIVEBORN and NeoBeat to provide both real-time feedback and debriefing with a focus on improving ventilation. The approach to the design of LIVEBORN will be described outside of this protocol in a design brief. The iterative development of LIVEBORN will be informed by formative research, usability evaluation, and integrated mHealth strategy design with feasibility testing.

4.1 FORMATIVE RESEARCH

4.1.1 Participant Recruitment

Prior to study activation, we will conduct outreach to relevant stakeholders and seek the necessary facility-level approvals. The three health facilities participating in formative research will be facilities in which the Kinshasa School of Public Health has previously conducted research with NeoBeat and a pilot version of LIVEBORN.

4.1.2 Participant Screening and Enrollment

Using a list of all midwives working at each facility, research staff will reach out to potential participants via telephone. LIVEBORN Study staff will assess basic eligibility criteria (e.g., provider of newborn resuscitation as well as history of clinical experience with NeoBeat or LIVEBORN). Potentially eligible participants will provide verbal informed consent in their preferred language by phone and will sign the informed consent form in person before the focus group discussion. The information sheet on participating in a research study during COVID-19 will be part of this informed consent process. Details of the informed consent process are included in Section 6 of this protocol.

4.1.3 Focus Group Discussions

LIVEBORN Study staff will hold one to two focus groups at each of three health facilities involving six to eight consenting midwives per focus group. Focus group discussions will last for approximately 60-90 minutes and will be conducted using a focus group discussion guide. Discussions will focus on attitudes and opinions about the use of a mobile health application to provide feedback both during and after newborn resuscitations. A video will be shown to help explain the concept of real-time guidance/feedback to ensure understanding among the participants. Discussions will also consider facility-specific challenges to newborn resuscitation as well as the facility environment for learning from clinical care. Focus group discussions will be audio-recorded.

DRC Ministry of Health recommendations for mitigating risks of COVID exposure in group gatherings will be followed, including best practices for mask usage and social distancing. These are detailed in the

information sheet on participating in a research study during COVID-19 and, at present, include the following additional study procedures:

1. No more than 24 hours prior to face-to-face focus group discussions, LIVEBORN Study staff will confirm the participant's appointment and perform telephone wellness screenings.
2. Upon a participant's arrival for the focus group discussion, LIVEBORN Study staff will rescreen the participant to confirm they have no symptoms, including no fever. Anyone who fails rescreening will be immediately isolated per health facility procedures and advised to seek care at a COVID testing site.
3. During face-to-face visits, LIVEBORN Study staff and participants will maintain a physical distance of three feet whenever possible, wear a facemask, and perform hand hygiene before and after face-to-face interaction.
4. Interactions will take place in an outdoor setting when possible.
5. LIVEBORN Study staff will ensure that the hospital policy for frequent cleaning and wiping of touched surfaces and objects with an approved disinfectant or disinfectant wipes are followed. This includes cleaning all chairs, tables or other equipment used in the focus group discussion, as well as disinfecting any surfaces that may be thought to be contaminated. LIVEBORN Study staff will ensure that an approved disinfectant is used such as a 1:10 dilution of bleach or 60-90% alcohol solution.

We will adapt these measures based on the prevailing Ministry of Health guidelines as needed.

4.2 Usability Evaluation

4.2.1 Participant Recruitment

Prior to study activation, we will seek the necessary facility-level approvals. We will conduct outreach with midwives at the hospital and invite them to participate in the simulated resuscitations.

4.2.2 Participant Screening and Enrollment

Using a list of all midwives who provide care to newborns at the time of birth at Bondo, research staff will reach out to potential participants via telephone. LIVEBORN Study staff will assess basic eligibility criteria. Potentially eligible participants will provide verbal informed consent in their preferred language by phone and will sign the informed consent form in person before participating in the usability evaluation. The information sheet on participating in a research study during COVID-19 will be part of this informed consent process. Details of the informed consent process are included in Section 6 of this protocol.

4.2.3 Usability Evaluation

We will evaluate the usability of real-time feedback and debriefing with LIVEBORN in two rounds of simulated resuscitations with consenting midwives. Midwives participating in the study will perform simulated resuscitations in each round (5-10 midwives per round). NeoNatalie Live (a high-fidelity manikin for low-resource settings developed by Laerdal Global Health [LGH]) will be used for all simulations. Resuscitation scenarios will be based on the HBB objective structured clinical exam for BMV, and will incorporate NeoBeat.

Prior to simulation, midwives will be oriented to the features and functions of LIVEBORN, NeoBeat, and NeoNatalie Live. All participating midwives will also complete a demographic survey.

Midwives will participate in simulated resuscitations in pairs: one midwife will participate as the newborn care provider, and the other midwife will participate as the observer who documents the

resuscitation using LIVEBORN. The midwife acting as the provider will resuscitate NeoNatalie Live with or without real-time guidance from LIVEBORN. During the resuscitation, the midwife acting as the observer will record all actions of the provider as well as the respiratory status of NeoNatalie Live. In resuscitations without real-time guidance, both the provider and observer will debrief using LIVEBORN after the simulation. All simulated resuscitations will be video-recorded along with the screen of the LIVEBORN app.

After the first round of simulated resuscitations, video recordings will be analyzed to identify design problems. Real-time guidance features, the LIVEBORN user interface and debriefing script will be refined accordingly. A second round of simulated resuscitations will be conducted with the refined version of LIVEBORN using the same procedures described above. Following both rounds of simulated resuscitations, midwives will complete the SUS, a validated scale for assessing app usability as well as the FIM.

4.2.4 Mitigating risks due to COVID-19

The day prior to any face-to-face interaction for this usability evaluation, study staff will screen all participants for COVID-19 symptoms via telephone.

For all face-to-face interactions, DRC Ministry of Health recommendations for mitigating risks of COVID exposure will be followed, including best practices for mask usage and social distancing. These are detailed in the information sheet on participating in a research study during COVID-19 and, at present, include the following additional study procedures:

1. No more than 24 hours prior to face-to-face simulated resuscitations, LIVEBORN Study staff will confirm the participant's appointment and perform telephone wellness screenings.
2. Upon a participant's arrival for the simulated resuscitations, LIVEBORN Study staff will rescreen the participant to confirm they have no symptoms, including no fever. Anyone who fails rescreening will be immediately isolated per health facility procedures and advised to seek care at a COVID testing site.
3. During face-to-face visits, LIVEBORN Study staff and participants will maintain a physical distance of three feet whenever possible, wear a facemask, and perform hand hygiene before and after face-to-face interaction.
4. Interactions will take place in an outdoor setting when possible.
5. LIVEBORN Study staff will ensure that the hospital policy for frequent cleaning and wiping of touched surfaces and objects with an approved disinfectant or disinfectant wipes are followed. This includes cleaning all chairs, tables or other equipment used in the simulated resuscitations, as well as disinfecting any surfaces that may be thought to be contaminated. LIVEBORN Study staff will ensure that an approved disinfectant is used such as a 1:10 dilution of bleach or 60-90% alcohol solution.

We will adapt these measures based on the prevailing Ministry of Health guidelines as needed.

4.3 Integrated Strategy Design with Feasibility Testing

4.3.1 Participant Recruitment

Prior to study activation, we will seek the necessary facility-level approvals. We will conduct outreach with midwives at the hospital and invite them to participate in the integrated strategy design with feasibility testing.

4.3.2 Participant Screening and Enrollment

Using a list of all midwives who provide care to newborns at the time of birth at the two identified health facilities, research staff will reach out to potential participants via telephone. LIVEBORN Study staff will assess basic eligibility criteria. Potentially eligible participants will provide verbal informed consent in their preferred language by phone and will sign the informed consent form in person before participating in integrated strategy design with feasibility testing. Details of the informed consent process are included in Section 6 of this protocol.

4.3.3 Design of Integrated mHealth Strategies for LIVEBORN

In collaboration with midwives at Binza-Delvaux and Kingasani health facilities in Kinshasa, we will design two integrated mHealth strategies: one for real-time guidance (at Binza-Delvaux) and one for debriefing (at Kingasani). We will use an established participatory research methodology called trials of improved practices (TIPs). TIPs engages stakeholders in the design of behavior change activities through an iterative process involving strategy development, small-scale testing and rapid analysis. TIPS allows participants to try a strategy in the clinical environment for a period of time and then provide feedback about its acceptability and feasibility for their context. Findings generated by this approach help researchers identify potential barriers, develop strategies to overcome those barriers, and eliminate or modify intervention components that are not feasible for participants.

Research staff will orient all participants to the LIVEBORN app and NeoBeat in short training sessions. At these training sessions, all midwives will complete a demographic survey. At Kingasani, research staff will also train midwives in how to debrief.

At both facilities, research staff will support head nurse midwives in developing initial strategies for both real-time guidance and debriefing that will include selection of the following elements:

- For both strategies,
  - Identification of bedside observers who will collect data on resuscitation practices using LIVEBORN such as colleagues, the supervisor or students
  - Selection of cases to observe (e.g., Convenience sample? High-risk? Certain shifts?)
  - System for cleaning and charging NeoBeat
  - System for charging and storing the observer's tablet
  - System for preparing resuscitation equipment before every birth, including NeoBeat and tablet
- For real-time guidance strategy,
  - System for charging and storing the guidance tablet
- For debriefing strategy,
  - Approach to debriefing, including when to debrief (immediately post-event or same shift), who should debrief (peer-to-peer or with supervisor support) and for what cases (well babies, neonatal deaths, stillbirths)

Midwives at Binza-Delvaux will implement the strategy for real-time guidance in the clinical environment, and midwives at Kingasani will implement the strategy for debriefing. During this small-scale test of approximately three weeks, research staff will conduct approximately weekly site visits to collect data through direct observations of the integrated strategy in clinical practice as well as a convenience sample of semi-structured interviews to evaluate the acceptability and feasibility of the strategy (~n=2-4 per visit). After small-scale testing, we will rapidly analyze the available data and

identify barriers to implementation of the strategy as well as potential solutions. In conjunction with head nurse midwives, we will design a refined strategy addressing these barriers. This strategy will again be implemented in a small-scale test lasting approximately three weeks with data collection per the first round. Data will be analyzed, and a new iteration of the strategy designed. This third strategy will again be evaluated in a small-scale test lasting approximately three weeks with data collected and analyzed per the first two rounds. After this iterative process, we will produce finalized strategies for feasibility testing.

4.3.4 Feasibility Testing

Following design of the integrated mHealth strategies for LIVEBORN, we will conduct a pilot feasibility test in both health facilities. For three months, midwives will incorporate the integrated mHealth strategy for LIVEBORN real-time guidance (Binza-Delvaux) or debriefing (Kingasani) into clinical care. At the end of the feasibility test, all midwives from both facilities will complete the SUS. Midwives in the real-time guidance facility will also complete the FIM. We will abstract the number of births at each facility during the feasibility test in order to calculate the percentage of observations conducted.

4.3.5 Mitigating risks due to COVID-19

The day prior to any face-to-face interaction for the training sessions, study staff will screen all training participants for COVID-19 symptoms via telephone. For all other research procedures and site visits/observations, study staff will rely on facility protocols that prevent sick staff from coming to work.

For all face-to-face interactions, DRC Ministry of Health recommendations for mitigating risks of COVID exposure will be followed, including best practices for mask usage and social distancing. At present, these include the following additional study procedures:

1. No more than 24 hours prior to face-to-face training sessions, LIVEBORN Study staff will confirm the participant's appointment and perform telephone wellness screenings.
2. Upon a participant's arrival for the study procedures, LIVEBORN Study staff will rescreen the participant to confirm they have no symptoms, including no fever. Anyone who fails rescreening will be immediately isolated per health facility procedures and advised to seek care at a COVID testing site.
3. During face-to-face visits, LIVEBORN Study staff and participants will maintain a physical distance of three feet whenever possible, wear a face mask, and perform hand hygiene before and after face-to-face interaction.
4. Interactions will take place in an outdoor setting when possible.
5. LIVEBORN Study staff will ensure that the hospital policy for frequent cleaning and wiping of touched surfaces and objects with an approved disinfectant or disinfectant wipes are followed. This includes cleaning all chairs, tables, or other equipment used during study procedures, as well as disinfecting any surfaces that may be thought to be contaminated. LIVEBORN Study staff will ensure that an approved disinfectant is used such as a 1:10 dilution of bleach or 60-90% alcohol solution.

We will adapt these measures based on the prevailing Ministry of Health guidelines as needed.

5 DATA MANAGEMENT AND ANALYSIS

5.1 FORMATIVE RESEARCH

5.1.1 Data Security and Management

All data will be kept confidential. No participant identifiers will be collected during formative research. Focus group discussions will be audio-recorded, transcribed and translated with secure transmission of translated documents to the co-investigators at UNC.

5.1.2 Sample Size

We will conduct up to nine focus group discussions of up to 10 participants each to identify barriers and facilitators to the use of LIVEBORN in clinical care, as well as preferences for real-time guidance and debriefing. Based on our prior conduct of focus group discussions around the development of an early version of LIVEBORN, this sample size will be adequate to identify salient issues for the further development of LIVEBORN.

5.1.3 Analysis

We will use rapid qualitative analysis to: 1) summarize individual responses in excel, 2) populate a matrix to identify themes, 3) sort and display visually and 4) outline findings to support refinement.[42]

5.2 USABILITY EVALUATION

5.2.1 Data Security and Management

All data will be kept confidential. No participant identifiers will be collected during the usability evaluation. Simulated resuscitations will be video-recorded. Surveys and video recordings will be securely transmitted by KSPH to UNC.

5.2.2 Sample Size

We will conduct up to 10 simulated resuscitations in each of two rounds of this usability evaluation. Design problems will be readily discoverable with this sample size as the participants will be multiple and homogenous, the task will be highly structured, and the feedback features will be newly developed.[43] This sample size will allow us to detect >85% of the usability problems available for discovery in each round.[44]

5.2.3 Analysis

Two investigators will independently review all video footage of midwives interacting with LIVEBORN to identify design problems after each round of simulated resuscitations. Additionally, we will score each participant's SUS and calculate the median SUS score to determine overall usability. We will also calculate the median FIM across participants.

5.3 INTEGRATED STRATEGY DESIGN AND FEASIBILITY TESTING

5.3.1 Data Security and Management

All data will be kept confidential. No participant identifiers will be collected during the TIPs or feasibility testing. All surveys and data will be securely transmitted by KSPH to UNC.

5.3.2 Sample Size
For the integrated strategy design and feasibility testing, we will engage with up to 50 midwives who are frontline providers in resuscitating newborns at Binza-Delvaux and Kingasani. During feasibility testing, each facility will implement their respective integrated strategy design for three months on a convenience sample of births (up to 300 births per facility). Based on our prior work with midwives in the development and refinement of LIVEBORN, this sample size will be adequate to evaluate both integrated mHealth strategies.

5.3.3 Analysis
After each round of small-scale testing, we will rapidly analyze data and generate a summary report to support refinement of the strategy.

For the feasibility testing, our primary outcome will be LIVEBORN observation feasibility. This will be defined as the percentage of resuscitations observed using LIVEBORN. We will consider LIVEBORN observation sufficiently feasible if observations are conducted for ≥ 50% of resuscitations. We will also evaluate the following secondary outcomes:

- LIVEBORN usability: score on the SUS. We will consider LIVEBORN sufficiently usable if the mean SUS score is >68.
- Real-time Guidance Feasibility: score on the FIM. We will consider the real-time guidance sufficiently feasible if the median FIM score is >12.
- Debriefing Feasibility: proportion of observed BMV episodes for which debriefing is completed. We will consider debriefing sufficiently feasible if ≥50% of observed BMV events have debriefing completed.

5.4 Dissemination of Findings
At the completion of the LIVEBORN Study, in accordance with National Institute of Health requirements, data produced by this research will be curated and documented, stripped of personal health information, and placed into the public domain where other groups can participate in its analysis and accelerate learning from it. Data will be available in commonly accessible formats with a data dictionary.

6 Ethical Considerations

6.1 Institutional Review Board Approval
The University of North Carolina (UNC) and Kinshasa School of Public Health (KSPH) Institutional Review Boards (IRBs) will approve this study protocol prior to the start of human subjects research. Given the progressive nature of this study, we will apply for IRB approval for formative research first. Informed by the results of our formative research, we will design the usability evaluation of LIVEBORN and apply for IRB approval with a modification to this study protocol. We will not initiate the usability evaluation until UNC and KSPH IRB approval have been granted for this aspect of the study. Informed by the results of our formative research and usability evaluation, we will develop the integrated mHealth strategy design with feasibility test and apply for IRB approval with an additional modification to this study protocol. We will not initiate the integrated strategy design with feasibility testing until UNC and KSPH IRB approval have been granted for this aspect of the study.

6.2 Formative Research

6.2.1 Informed Consent

To protect participant confidentially, we will conduct all research-related discussions and informed consent procedures on a private phone call or in a private room. If a private room is not available, a designated area far enough away from other people such that they cannot hear the conversation will be used. We will reach out to potential participants by phone for consent to participate in a focus group discussion. We will obtain verbal informed consent in French, the native language, using the IRB-approved telephone script/consent form. We will explain the purpose and procedures for the focus group discussion, including audio-recording, transcription and translation of the discussion. Additionally, we will review the information sheet on participating in a research study during COVID-19. We will give adequate opportunity for each potential participant to understand the consent form and ask questions. Fair balance will be maintained while describing the risks and benefits of participation in the study. No undue pressure will be placed on the potential participant to enroll in the study. After the potential participant has heard the consent form for participation in the study, and agrees to commit to participating, the research staff will sign the form. Participants will then be asked to also sign the consent form in person when they come to the focus group discussion. The research staff will retain signed copies of the form. Each midwife will receive 10USD to cover their travel for participation in the focus group discussion.

6.2.2 Potential Risks to Participants

There are minimal risks to participants in this study. There is a small risk of breach of confidentiality through participation in a focus group discussion. For example, discussion regarding barriers to resuscitation may include specific criticisms of the health facility or other workplace concerns. Breach of confidentiality will be minimized by audio-recording discussions without any identifying information, and encouraging participants to keep the group's discussion confidential.

6.2.3 Potential Benefits to Participants

There are no benefits to participants in this study.

6.3 Usability Evaluation

6.3.1 Informed Consent

To protect participant confidentially, we will conduct all research-related discussions and informed consent procedures on a private phone call or in a private room. If a private room is not available, a designated area far enough away from other people such that they cannot hear the conversation will be used. We will reach out to potential participants by phone for consent to participate in the usability evaluation. We will obtain verbal informed consent in French, the native language, using the IRB-approved telephone script/consent form. We will explain the purpose and procedures for the usability evaluation, including video recording of the simulated resuscitations. Additionally, we will review the information sheet on participating in a research study during COVID-19. We will give adequate opportunity for each potential participant to understand the consent form and ask questions. Fair balance will be maintained while describing the risks and benefits of participation in the study. No undue pressure will be placed on the potential participant to enroll in the study. After the potential participant has heard the consent form for participation in the study, and agrees to commit to participating, the research staff will sign the form. Participants will then be asked to also sign the consent form in person when they come to the usability evaluation. The research staff will retain signed

copies of the form. Each midwife will receive 10USD to cover her travel for participation in the usability evaluation.

6.3.2 Potential Risks to Participants

There are minimal risks to participants in this study. There is a small risk of embarrassment or emotional distress for the provider receiving feeding from LIVEBORN regarding her performance during the simulated resuscitation. This risk is minimized by language in the app designed to promote a culture of inquiry rather than one of blame. There is also a risk of breach of confidentiality. Data will be collected through video footage of the simulated resuscitations as well as surveys. All study records will identify midwives by a unique study number. The only link between the midwife's name and the study number will be the paper study log. Data from this log will be maintained locally and will not be transmitted to the co-investigators at UNC. The log will be kept in a secure, locked location.

6.3.3 Potential Benefits to Participants

There are no benefits to participants in this study.

6.4 INTEGRATED STRATEGY DESIGN AND FEASIBILITY TESTING

6.4.1 Informed Consent

To protect participant confidentially, we will conduct all research-related discussions and informed consent procedures on a private phone call or in a private room. If a private room is not available, a designated area far enough away from other people such that they cannot hear the conversation will be used. We will reach out to potential participants by phone for consent to participate in the integrated strategy design and feasibility testing. We will obtain verbal informed consent in French, the native language, using the IRB-approved telephone script/consent form. We will explain the purpose and procedures for the integrated strategy design and feasibility testing. We will give adequate opportunity for each potential participant to understand the consent form and ask questions. Fair balance will be maintained while describing the risks and benefits of participation in the study. No undue pressure will be placed on the potential participant to enroll in the study. After the potential participant has heard the consent form for participation in the study, and agrees to commit to participating, the research staff will sign the form. Participants will then be asked to also sign the consent form in person at the start of study procedures. The research staff will retain signed copies of the form. Each midwife will receive 10USD to cover her travel for participation in the initial training in the LIVEBORN app and NeoBeat.

6.4.2 Potential Risks to Participants

There are minimal risks to participants in this study. There is a small risk of embarrassment or emotional distress for the provider receiving feedback from LIVEBORN regarding her performance during a resuscitation. This risk is minimized by language in the app designed to promote a culture of inquiry rather than one of blame. There is also a risk of breach of confidentiality. Data will be collected through surveys, structured interviews, and direct observation. All study records will identify midwives by a unique study number. The only link between the midwife's name and the study number will be the paper study log. Data from this log will be maintained locally and will not be transmitted to the co-investigators at UNC. The log will be kept in a secure, locked location.

6.4.3 Potential Benefits to Participants

There are no benefits to participants in this study.

7 Study Organization, Coordination and Administration

7.1 Division of Responsibilities

The study team will be composed of personnel from four institutions: UNC, KSPH, LGH, and Jhpiego. Responsibilities of each organization are detailed below, with roles for specific investigators detailed in the table.

7.1.1 UNC

UNC will coordinate all communication between the partner institutions and lead the study. This includes leading the development of the study protocol and manual of operations, as well as data analyses and reporting. UNC will develop the data management system and oversee data cleaning.

7.1.2 KSPH

KSPH will lead the execution of the study protocol. They will hire and train all staff for the study. They will recruit and enroll all participants in the health facilities. They will conduct all facility preparatory activities. They will coordinate and supervise data collection procedures to ensure accurate data collection and expedient data transmission and error resolution. They will administer all questionnaires to participants and lead all focus group discussions. They will conduct study monitoring and quality assurance activities. They will ensure participant confidentiality and safety, and report any unanticipated problems, adverse events, and serious adverse events.

7.1.3 LGH

LGH will lead the technical development of LIVEBORN in close conjunction with the PI, Dr. Patterson. LGH will manage the cloud server that houses data from the LIVEBORN app. They will provide all data collected on the cloud server during the trial to UNC for analysis. They will also provide technical advice to KSPH to support LIVEBORN.

7.1.4 Jhpiego

Jhpiego will advise on intervention design and sustainability, including participating in the design of LIVEBORN and development of the study protocol and manual of operations.

Role of Key Personnel

Investigators	Affiliation	Role
Jacquelyn Patterson (PI)	UNC	Lead development of study materials, analysis, publication<br>Coordinate communication among partner organizations
Eric Mafuta (Co-I)	KSPH	Partner in development/implementation/evaluation<br>Conduct focus group discussions
Ben Chi (Co-I)	UNC	Mentor ESIs in developing study materials, analysis, publication
Carl Bose (Co-I)	UNC	Mentor ESIs in mHealth intervention development<br>Facilitate conduct of study in the DRC
Helge Myklebust (Co-I)*	LGH	Partner in mHealth intervention development<br>Oversee development and iterative refinement of LIVEBORN
Antoinette Tshefu (Co-I)*	KSPH	Mentor ESIs in study implementation<br>Facilitate conduct of study in the DRC
Patricia Gomez (Co-I)*	Jhpiego	Partner in mHealth intervention development

	Advise on intervention sustainability/scale-up
*Consortium PI; ESI=early stage investigator, DRC=Democratic Republic of the Congo	

7.2 Communication

To ensure the adequacy of continuous communication among key personnel in this study, we will establish a study steering committee made up of the PI and all Co-Is, and led by Dr. Patterson (the PI). The committee will rely on telephone calls, email and internet-based conferencing such as Zoom for communication. During the development of the study design as well as implementation of the study protocol, we will hold at least once monthly conference calls. In addition to the PI and Co-Is, relevant personnel from each collaborating organization will join as needed (e.g., the technical leads from LGH).

7.3 Study Timeline

We anticipate completing this study in 24 months, with the timeline of activities detailed as follows.

Timeline of R21 activities: 9/9/2020 through 8/31/2022

**Activity**	**Year 1**				**Year 2**			
	**Q1 (Sept-Nov '20)**	**Q2 (Dec '20-Feb '21)**	**Q3 (Mar-May '21)**	**Q4 (June-Aug '21)**	**Q1 (Sept-Nov '21)**	**Q2 (Dec '21-Feb '22)**	**Q3 (Mar-May '22)**	**Q4 (June-Aug '22)**
Technical development	(start Oct)				(end Sept)			
Design brief								
Formative research protocol/IRB								
Concept development								
Implementation of formative research/data analysis		(Jan/Feb)						
Usability evaluation protocol/IRB/MOP								
Implementation of usability evaluation/data analysis (round 1/2)				(May)	(Sept)			
Design/feasibility testing protocol/IRB/MOP								
Design of integrated mHealth strategies					(start Oct)	(end Dec)		
Feasibility testing						(start Jan)	(end Mar)	
Final analysis								
Submission of R33 transition package								
R33 trial protocol development								

8 References

1. World Health Organization Fact Sheet. Newborns: reducing mortality. 2019; https://www.who.int/news-room/fact-sheets/detail/newborns-reducing-mortality. Accessed October 29, 2019.
2. Kamath-Rayne BD, Griffin JB, Moran K, Jones B, Downs A, McClure EM, Goldenberg RL, Rouse D, Jobe AH. Resuscitation and Obstetrical Care to Reduce Intrapartum-Related Neonatal Deaths: A MANDATE Study. *Maternal and child health journal.* 2015;19(8):1853-1863

3. Singh K, Brodish P, Speizer I, Barker P, Amenga-Etego I, Dasoberi I, Kanyoke E, Boadu EA, Yabang E, Sodzi-Tettey S. Can a quality improvement project impact maternal and child health outcomes at scale in northern Ghana? *Health Res Policy Syst.* 2016;14(1):45.PMC4910198
4. White AE, Ng HX, Ng WY, Ng EK, Fook-Chong S, Kua PH, Ong ME. Measuring the effectiveness of a novel CPRcard feedback device during simulated chest compressions by non-healthcare workers. *Singapore medical journal.* 2017;58(7):438-445.PMC5523097
5. Ersdal HL, Mduma E, Svensen E, Perlman JM. Early initiation of basic resuscitation interventions including face mask ventilation may reduce birth asphyxia related mortality in low-income countries: a prospective descriptive observational study. *Resuscitation.* 2012;83(7):869-873
6. Moshiro R, Perlman JM, Kidanto H, Kvaloy JT, Mdoe P, Ersdal HL. Predictors of death including quality of positive pressure ventilation during newborn resuscitation and the relationship to outcome at seven days in a rural Tanzanian hospital. *PloS one.* 2018;13(8):e0202641.PMC6097682 Foundation for Acute Medicine for work in Tanzania. Haydom Lutheran Hospital has received research grants from the Laerdal Foundation for Acute Medicine. This does not alter our adherence to PLOS ONE policies on sharing data and materials.
7. Linde JE, Perlman JM, Oymar K, Schulz J, Eilevstjonn J, Thallinger M, Kusulla S, Kidanto HL, Ersdal HL. Predictors of 24-h outcome in newborns in need of positive pressure ventilation at birth. *Resuscitation.* 2018;129:1-5
8. Dol J, Campbell-Yeo M, Murphy GT, Aston M, McMillan D, Richardson B. The impact of the Helping Babies Survive program on neonatal outcomes and health provider skills: a systematic review. *JBI database of systematic reviews and implementation reports.* 2018;16(3):701-737
9. Matendo R, Engmann C, Ditekemena J, Gado J, Tshefu A, Kinoshita R, McClure EM, Moore J, Wallace D, Carlo WA, Wright LL, Bose C. Reduced perinatal mortality following enhanced training of birth attendants in the Democratic Republic of Congo: a time-dependent effect. *BMC Med.* 2011;9:93.3171324
10. Binder C, Schmolzer GM, O'Reilly M, Schwaberger B, Urlesberger B, Pichler G. Human or monitor feedback to improve mask ventilation during simulated neonatal cardiopulmonary resuscitation. *Archives of disease in childhood Fetal and neonatal edition.* 2014;99(2):F120-123
11. Fuerch JH, Yamada NK, Coelho PR, Lee HC, Halamek LP. Impact of a novel decision support tool on adherence to Neonatal Resuscitation Program algorithm. *Resuscitation.* 2015;88:52-56
12. Sawyer T, Motz P, Schooley N, Umoren R. Positive pressure ventilation coaching during neonatal bag-mask ventilation: A simulation-based pilot study. *Journal of neonatal-perinatal medicine.* 2019;12(3):243-248
13. Garcia-Suarez M, Mendez-Martinez C, Martinez-Isasi S, Gomez-Salgado J, Fernandez-Garcia D. Basic Life Support Training Methods for Health Science Students: A Systematic Review. *International journal of environmental research and public health.* 2019;16(5).PMC6427599
14. Skare C, Boldingh AM, Kramer-Johansen J, Calisch TE, Nakstad B, Nadkarni V, Olasveengen TM, Niles DE. Video performance-debriefings and ventilation-refreshers improve quality of neonatal resuscitation. *Resuscitation.* 2018;132:140-146
15. Skare C, Calisch TE, Saeter E, Rajka T, Boldingh AM, Nakstad B, Niles DE, Kramer-Johansen J, Olasveengen TM. Implementation and effectiveness of a video-based debriefing programme for neonatal resuscitation. *Acta anaesthesiologica Scandinavica.* 2018;62(3):394-403
16. Huang J, Tang Y, Tang J, Shi J, Wang H, Xiong T, Xia B, Zhang L, Qu Y, Mu D. Educational efficacy of high-fidelity simulation in neonatal resuscitation training: a systematic review and meta-analysis. *BMC medical education.* 2019;19(1):323.PMC6716944
17. Dempsey E, Pammi M, Ryan AC, Barrington KJ. Standardised formal resuscitation training programmes for reducing mortality and morbidity in newborn infants. *Cochrane Database Syst Rev.* 2015(9):CD009106

18. Agravat N. *mHealth and Neonatal Resuscitation: A Review of Interventions, Approaches and Applications.* Washington, DC: mHealth Alliance;2013.
19. Umoren R, Bucher S, Mairami A, Purkayastha S, Bresnahan B, Ezeaka C, Esamai F, Paton C. eHBB/mHBS powered by DHIS2: Mobile Virtual Reality Newborn Provider Training in Helping Babies Breathe. Paper presented at: 2019 International Meeting on Simulation in Healthcare; January 27, 2019, 2019; San Antonio, TX.
20. ClinicalTrials.gov. Development of a Mobile Application for HBB Prompt Study (HBB-Prompt). 2018; https://clinicaltrials.gov/ct2/show/NCT03577054. Accessed November 4, 2019.
21. Trevisanuto D, Bertuola F, Lanzoni P, Cavallin F, Matediana E, Manzungu OW, Gomez E, Da Dalt L, Putoto G. Effect of a Neonatal Resuscitation Course on Healthcare Providers' Performances Assessed by Video Recording in a Low-Resource Setting. *PloS one.* 2015;10(12):e0144443.PMC4684235
22. Somannavar MS, Goudar SS, Revankar AP, Moore JL, McClure EM, Destefanis P, DeCain M, Goco N, Wright LL. Evaluating time between birth to cry or bag and mask ventilation using mobile delivery room timers in India: the NICHD Global Network's Helping Babies Breathe Trial. *BMC pediatrics.* 2015;15:93-015-0408-0406
23. Skare C, Boldingh AM, Nakstad B, Calisch TE, Niles DE, Nadkarni VM, Kramer-Johansen J, Olasveengen TM. Ventilation fraction during the first 30s of neonatal resuscitation. *Resuscitation.* 2016;107:25-30
24. National Academies of Sciences E, and Medicine. *Improving quality of care in low- and middle-income countries: Workshop summary.* Washington, DC: The National Academies Press; 2015.
25. Mejia-Guevara I, Zuo W, Bendavid E, Li N, Tuljapurkar S. Age distribution, trends, and forecasts of under-5 mortality in 31 sub-Saharan African countries: A modeling study. *PLoS Med.* 2019;16(3):e1002757.PMC6413894
26. WHO. Sustainable Development Goals. https://www.who.int/gho/publications/mdgs-sdgs/MDGs-SDGs2015_chapter4.pdf?ua=1. Accessed November 4, 2019.
27. *Plan D'Action Chaque Nouveau-NE, 2018-2020.* 2018.
28. Morley CJ. Monitoring Neonatal Resuscitation: Why Is It Needed? *Neonatology.* 2018;113(4):387-392
29. Park JH, Shin SD, Ro YS, Song KJ, Hong KJ, Kim TH, Lee EJ, Kong SY. Implementation of a Bundle of Utstein Cardiopulmonary Resuscitation Programs to Improve Survival Outcomes after Out-of-Hospital Cardiac Arrest in a Metropolis: A Before and After Study. *Resuscitation.* 2018
30. Tobase L, Peres HHC, Tomazini EAS, Teodoro SV, Ramos MB, Polastri TF. Basic life support: evaluation of learning using simulation and immediate feedback devices1. *Revista latino-americana de enfermagem.* 2017;25:e2942.PMC5706606
31. Weston BW, Jasti J, Lerner EB, Szabo A, Aufderheide TP, Colella MR. Does an individualized feedback mechanism improve quality of out-of-hospital CPR? *Resuscitation.* 2017;113:96-100
32. Gelbart B, Hiscock R, Barfield C. Assessment of neonatal resuscitation performance using video recording in a perinatal centre. *Journal of paediatrics and child health.* 2010;46(7-8):378-383
33. Craig P, Dieppe P, Macintyre S, Michie S, Nazareth I, Petticrew M. Developing and evaluating complex interventions: the new Medical Research Council guidance. *BMJ (Clinical research ed).* 2008;337:a1655.PMC2769032
34. O'Donnell CP, Kamlin CO, Davis PG, Morley CJ. Ethical and legal aspects of video recording neonatal resuscitation. *Archives of disease in childhood Fetal and neonatal edition.* 2008;93(2):F82-84
35. Shivananda S, Twiss J, El-Gouhary E, El-Helou S, Williams C, Murthy P, Suresh G. Video recording of neonatal resuscitation: A feasibility study to inform widespread adoption. *World journal of clinical pediatrics.* 2017;6(1):69-80.PMC5296632

36. Kardong-Edgren SE, Oermann MH, Odom-Maryon T, Ha Y. Comparison of two instructional modalities for nursing student CPR skill acquisition. *Resuscitation.* 2010;81(8):1019-1024
37. Isbye DL, Hoiby P, Rasmussen MB, Sommer J, Lippert FK, Ringsted C, Rasmussen LS. Voice advisory manikin versus instructor facilitated training in cardiopulmonary resuscitation. *Resuscitation.* 2008;79(1):73-81
38. Magee MJ, Farkouh-Karoleski C, Rosen TS. Improvement of Immediate Performance in Neonatal Resuscitation Through Rapid Cycle Deliberate Practice Training. *Journal of graduate medical education.* 2018;10(2):192-197.PMC5901799 study was presented as a poster at the 8th International Pediatric Simulation Symposia and Workshop, Glasgow, Scotland, May 9-11, 2016; as a poster at the Pediatric Academic Societies Meeting, San Francisco, California, May 6-9, 2017; and as a platform presentation at the 9th International Pediatric Simulation Symposia and Workshop, Boston, Massachusetts, June 1-3, 2017.
39. Sawyer T, Sierocka-Castaneda A, Chan D, Berg B, Lustik M, Thompson M. Deliberate practice using simulation improves neonatal resuscitation performance. *Simulation in healthcare : journal of the Society for Simulation in Healthcare.* 2011;6(6):327-336
40. Brooke J. A "quick and dirty" usability scale. In: Thomas B, Weerdmester B, MecClelland I, eds. *Usability Evaluation in Industry.* London: Taylor and Francis; 1996:189-194.
41. Weiner BJ, Lewis CC, Stanick C, Powell BJ, Dorsey CN, Clary AS, Boynton MH, Halko H. Psychometric assessment of three newly developed implementation outcome measures. *Implementation science : IS.* 2017;12(1):108.PMC5576104
42. Gale RC, Wu J, Erhardt T, Bounthavong M, Reardon CM, Damschroder LJ, Midboe AM. Comparison of rapid vs in-depth qualitative analytic methods from a process evaluation of academic detailing in the Veterans Health Administration. *Implementation science : IS.* 2019;14(1):11.PMC6359833
43. Sauro J, Lewis JR. *Quantifying the User Experience: Practical Statistics for User Research.* San Francisco, CA, USA: Morgan Kaufmann Publishers Inc.; 2012.
44. Nielsen J, Landauer TK. A mathematical model of the finding of usability problems. Paper presented at: Proceedings of ACM INTERCHI'93 Conference; April 24-29, 1993; Amsterdam, The Netherlands.
