# Supplementary material for: Mobile health supported real-time guidance and debriefing for newborn resuscitation: A pilot study of LIVEBORN feedback
Source: PLoS One. 2026 Jan 8;21(1):e0310606. doi: 10.1371/journal.pone.0310606 (PMC12782419; doi:10.1371/journal.pone.0310606)
Supplement: Attachment — Submitted filename: Review PONE-D-24-35966_2025 03 23.pdf [file pone.0310606.s002.pdf]

PlosOne PONE-D-24-35966

Thank you for the opportunity to review the manuscript titled, *Mobile Health Supported Real-Time Guidance and Debriefing for Newborn Resuscitation: A Pilot Study of LIVEBORN Feedback.* This paper presents new evidence for application of a real-time guidance tool for neonatal resuscitation in LMICs. The need for improving neonatal outcomes in LMICs and supporting midwives during reception of the newborn reflects the importance of LIVEBORN. There are a few suggestions for the authors to consider before publication is considered, as follows:
Suggest minor editing to improve sentence structure and punctuation.

Introduction

p. 4. The introduction states "… *HBB training are often not sustained due to a decline in knowledge and skill over time*." This sentence is not clear in relation to who HBB training is relevant to. For example, are you referring to midwives? Why is knowledge and skill related to BMV declining over time? Are T-piece devices available in these settings?

p. 5. What type of mixed methods approach was used? Please define and provide a reference to enhance this section.

Materials and Methods

Study Design and Participants

p. 5. Sampling strategy should be noted here. Was purposive sampling of participants used based on midwives who have specialised knowledge of resuscitation?

p. 5. What was the target recruitment number of midwives?

p. 5. A section on the study sites could have been added here. For example, how many births were recorded at each site per year? What level of capability/facilities available to support neonatal care at each site? Level of staffing at each facility.

Description of LIVEBORN Feedback

p. 6. More information needed on LIVEBORN usability would be beneficial. Does the observer manually type (free text) in observations during a real-time resuscitation or are there pre-populated fields and drop-down boxes to minimise data entry errors?

p. 6-7. Is the ongoing practice of prolonged suctioning linked to a *decline in knowledge and skills over time* (from p. 4*)?* It is not clear why this practice remains despite simulation training.

Training in Use of LIVEBORN Feedback

p. 7. You emphasized the importance of creating a *culture of improving care*. This sentence fails to acknowledge the importance of health professionals being reflexive practitioners. Suggest some mention of reflexive practice, which in turn influences culture and the provision of care.

Strategy Design and Refinement

More information on the methodology, Trials of Improved Practices, would enhance this section. Currently, this methodology is not well explained or justified when using a MM approach.

p. 8. "*using a participatory research methodology called Trials of Improved Practices (TIPS)*". This sentence requires editing to improve readability. The first word, using, is not capitalised.

If over 50% of facility births were targeted, how many births does this equate to?

During the testing phase, what barriers were identified to implementation of the strategy? What changes were made in response to identified barriers?

Qualitative Evaluation

p. 10. Convenience sampling was used. Would purposive sampling be relevant considering midwives possess specialist knowledge on resuscitation?

pp. 10-11. Were all midwives who participated in the focus groups equal in terms of position status? It is not clear here if any midwife participants had higher responsibilities than the others with the potential for power imbalances existing during the focus groups.

Results

Strategy Design and Refinement

p. 12. Environmental health services staff have not been mentioned prior to now. Who are these staff? A description and recruitment strategy would be beneficial.

p. 12. " … the *nurse* assigned to acute care ensured that it was charged". Apologies if I missed this, but aren't the participants midwives? If so, why are nurses included in the results? Is this nurse a research study nurse? Nurses in the acute care should be included in the methods if involved in the study. Were they recruited and PICF provided?

p. 12. Table 1 Who are skilled birth attendants? Are these midwives? There is some inconsistency with titles if they are the same. If not, please clarify each role.

p. 12. Table 1 **Who observes births** and **Which births are observed** are questions. Insert question marks or reword, for example, *Birth observer* and *Observed birth type*. Similarly, please edit questions in Table 2.

p. 12. Strategy design involving recruitment target for midwives or observed resuscitation activities should be stated in methods (recruitment).

p. 13. Legend requires punctuation between numbers 1-5.

p. 13. *Checklist was found to be burdensome.* In view of these burdensome features, what modifications have been made for the RCT?

p. 18. Nurse trainees and non-clinical staff as intra-facility observers were introduced in the discussion. These staff should have been introduced in the methods section, including recruitment strategies.

p. 18. Data documented shortly after the resuscitation highlights a limitation with LIVEBORN. What adaptations have been made to ensure post-resuscitation documentation is minimised or avoided for the proposed RCT?

p. 19. You state, "*beyond feasibility, instilling confidence in the guidance may be key to changing behavior*". Might ongoing professional education of midwives be key to improving resuscitation techniques and hence, their behaviour?

p. 20. Edit out additional comma, "Given prior data in these health facilities indicating that providers fail to initiate ventilation in 80% of the cases in which it is indicated,, …"

p. 20. Additional cases were added to the debrief and results: Wasn't there an eligibility for BMV debrief to ensure additional and irrelevant cases did not conflate your results?

p. 21. The use of mixed methods for analysis was not adequately linked to the theoretical approach of TIPS. At what points were the quantitative and qualitative data merged? Did the results of the quantitative data inform the topics for focus group discussion?

p.21. Limitations are clearly presented and provide direction for future implementation of LIVEBORN.
